# A History of Mild Traumatic Brain Injury Affects Peripheral Pulse Oximetry during Normobaric Hypoxia

**DOI:** 10.3389/fneur.2016.00149

**Published:** 2016-09-21

**Authors:** Leonard A. Temme, Paul St. Onge, Joseph Bleiberg

**Affiliations:** ^1^U.S. Army Aeromedical Research Laboratory, Fort Rucker, AL, USA; ^2^Laulima Government Solutions, LLC, Orlando, FL, USA; ^3^National Intrepid Center of Excellence, Walter Reed National Military Medical Center, Bethesda, MD, USA

**Keywords:** mild traumatic brain injury, normobaric hypoxia, pulse oximetry, Functional Impairment Tester, oculometric, stress

## Abstract

**Introduction:**

Physiological and emotional stressors increase symptoms of concussion in recently injured individuals and both forms of stress-induced symptoms in people recovering from mild traumatic brain injury (mTBI), but who are asymptomatic when not stressed or are at rest.

**Methods:**

Healthy asymptomatic adults (25.0 ± 5.1 years) with a history of mTBI (*n* = 36) and matched healthy controls (HC) (*n* = 36) with no mTBI history were exposed to three levels of normobaric hypoxic stress generated with the Reduced Oxygen Breathing Device (ROBD) (Environics, Inc., Tollande, CT, USA), which reduced the percent O_2_ by mixing sea level air with nitrogen. The ROBD reduced the percent O_2_ in the breathable air from the normal 21% to 15.5% O_2_, 14% O_2_, and 13% O_2_. Under these conditions: (a) a standard pulse oximeter recorded peripheral oxygen saturation (SpO_2_) and pulse rate (beats per minute) and (b) the Functional Impairment Tester (FIT) (PMI, Inc., Rockville, MD, USA) recorded saccadic velocity and pupillary response dynamics to a brief light flash.

**Results:**

For all three hypoxic stress conditions, the mTBI group had significantly higher SpO_2_ during the final minute of exposure than did the controls [*F*(2.17,151.8) = 5.29, *p* < 0.001, η^2^ = 0.852] and the rate of SpO_2_ change over time was significantly shallower for the mTBI than for the controls [*F*(2.3,161.3) = 2.863, *p* < 0.001, η^2^ = 0.569], Greenhouse–Geisser corrected. Overall, mTBI had lower pulse rate but the difference was only significant for the 14% O_2_ condition. FIT oculomotor measures were not sensitive to group differences. When exposed to mild or moderate normobaric hypoxic stress (15% O_2_): (1) SpO_2_ differences emerged between the mTBI and matched HC groups, (2) heart rate trended lower in the mTBI group, and (3) FIT measures were not sensitive to group differences.

**Conclusion:**

A relatively minor hypoxic challenge can reveal measurable differences in SpO_2_ and heart rate in otherwise asymptomatic individuals with a history of mTBI.

## Introduction

Physiological or emotional stressors can increase symptoms of mild traumatic brain injury (mTBI) in recently injured individuals, and either type of stress can induce symptoms in individuals with mTBI who are asymptomatic when not stressed (1). For example, compared to groups of normal controls, mTBI groups showed higher heart rate (2) and decreased heart rate variability (HRV) (3, 4) as well as reduced cerebrovascular reactivity and lower end-tidal carbon dioxide (PETCO_2_) trends (5, 6) in response to experimental stress. The stressors used in these experiments included exercise (3, 7), repeated breath holds and hyperventilation (5, 6), psychosocial stress (8), and mental arithmetic under conditions of noise and bright lights (2).

Hypoxia is a physiological stressor that may be readily encountered in daily life and can be relatively easily and reliably generated in a controlled laboratory setting, thus permitting precise quantification of the hypoxic physiological stressor and its effects on the volunteer. Ewing et al. (9), using an altitude chamber to generate altitude-related hypoxia, exposed a group of controls and a group of asymptomatic, concussed individuals to a simulated altitude of 3,800 m (nearly 12,500 ft). These authors reported finding a significant cognitive performance deficit in the brain-injured group at altitude, a difference which disappeared when the subjects were returned to ground level. Temme et al. (10, 11) extended the Ewing et al. study using normobaric hypoxia (NH) rather than an altitude chamber to generate hypoxic stress. NH mixes nitrogen (N_2_) with mean sea level (MSL) air to reduce the percentage of inspired oxygen from the normal sea level value of 21%, a technique that is widely used in current respiratory physiology. Temme et al. found (1) at MSL, there were no differences between the mTBI and control groups in cognitive performance; (2) exposure to NH with 15.5% O_2_, 14% O_2_, and 13% O_2_ (approximately equivalent to breathing air encountered at 8,000, 12,000, and 14,000 ft above MSL, respectively) resulted in a decrease in cognitive performance for both groups; (3) when hypoxic, the visual short-term memory of the mTBI group was significantly poorer than that of the control group; and (4) there was no difference between the groups on returning to 21% O_2_, normal MSL air. These studies support the idea that NH is an effective stressor that can uncover symptoms of mTBI in individuals who are asymptomatic when unstressed.

Eye movements are a sensitive indicator of mTBI and can provide a great deal of objective diagnostic information about the concussed brain (12–15). Recently, instrumentation to measure a pupil’s response to light flashes, and the eye’s ballistic movements, saccades, was integrated into a device designed to screen for physiological impairments due to fatigue, sleep deprivation, drug and alcohol intoxication, and other physiological stressors (16–21). The device, the Functional Impairment Tester (FIT), measures ocular motility along four dimensions: pupil diameter in the dark, the latency of the pupil’s response to flashes of light, the pupil’s amplitude of constriction, and the eye’s saccadic velocity (17, 18, 20). The literature reports that these dimensions of the pupil, i.e., the pupillary light reflex (22) and the eye’s saccadic behavior (12), can be affected by mTBI. Moreover, FIT measures have demonstrated sensitivity to hypoxia (17). To date, however, the FIT has not been established as an assessment or screening tool for concussion or mTBI. Since the behavior of the eye is a standard clinical indicator of brain injury and since vision deteriorates with hypoxia, the FIT was selected as a potential non-subjective oculomotor test to differentiate between controls and mTBI subjects during NH stress. The present study extends the Temme et al. (10) cognitive finding by evaluating response differences to NH between the mTBI group and the matched healthy control (HC) group in the physiological measures of peripheral oxygen saturation (SpO_2_) and pulse rate, as well as oculometric.

## Materials and Methods

The volunteers and the procedures used in this study were described previously in detail by Temme et al. (10). The study was reviewed, approved, and conducted in accordance with all Federal and State laws, regulations, and standards of practice, and in accordance with all Department of Defense and U.S. Army regulations and procedures. The research protocol was approved by the Chesapeake Institutional Review Board, the U.S. Army Aeromedical Research Laboratory Human Use Committee, and the U.S. Army Medical Research and Materiel Command Human Subjects Research Review Board. The study was determined to pose a greater than minimal risk to the subjects; and as part of risk mitigation, the NH stress conditions were presented in an increasing sequence of severity with each subject being observed at a lower stress condition before being exposed to a greater stress.

### Experimental Design

The experimental design was a two-factor mixed model with two levels of the between-subject variable and five levels of the within-subject variable. The between-subject variable was mTBI history, with one group of subjects having an mTBI history and one group without. The within-subject variable was percent O_2_ of the inspired air; all subjects were exposed to five levels, in the following order: 21% O_2_ Baseline, 15.5% O_2_, 14% O_2_, 13% O_2_, and 21% O_2_ Post NH Stress.

### Human Subject Volunteers

All subjects were screened by the study physician to ensure compliance with the following exclusion criteria: pregnancy; history of drug or alcohol abuse; depression; bipolar disorder; schizophrenia; problems with the heart, kidney, liver, asthma, strokes, mini-strokes, or poor leg circulation; any ongoing medical problems; current or past neurological problems such as seizures, epilepsy, or dementia; post-traumatic headache; current concentration and/or memory problems caused by a head injury; loss of consciousness >30 min at the time of injury; or post-traumatic amnesia >24 h at the time of injury.

For inclusion into the mTBI group, subjects met criteria that closely followed the criteria of the American Congress of Rehabilitation Medicine (23); a duration of a loss of consciousness of no more than 30 min at the time of the TBI, a duration of post-traumatic amnesia of no more than 24 h, a Glasgow Coma Score of 13–15 (24), and a clinical history consistent with the diagnosis of mTBI. All members of the control group denied any history of concussion or brain injury, and each HC was matched with a member of the mTBI group on the basis of gender, age, body mass index (BMI), and smoking behavior (either 0 to 9 cigarettes a day or 10 or more cigarettes a day). Each group consisted of 9 women and 27 men for a total of 36 subjects per group. Each member of the matched pair of mTBI and HC was tested within a week of each other in order to minimize possible inadvertent differences due to drift in instrumentation, methodology, procedures, and personnel changes.

Table 1 provides the mean and SD by group of age, weight, height, BMI, resting systolic and diastolic blood pressure, pulse rate, and respiration rate. An analysis of variance showed that the mTBI and HC groups did not differ statistically along any of the parameters in Table 1; the probabilities (*p*-values) for these comparisons ranged from a high of 0.817 to a low of 0.180, all larger than 0.05.

**Table 1:** **Summary of the characteristics of the mTBI and HC groups (*n* = 36/group)**.

	Group	M ± SD		Group	M ± SD
Age (years)	mTBI	25.25 ± 5.42	Systolic (mmHg)	mTBI	120.14 ± 14.79
	HC	24.89 ± 4.94		HC	120.94 ± 17.62
Weight (kg)	mTBI	90.75 ± 29.42	Diastolic (mmHg)	mTBI	73.89 ± 9.79
	HC	95.70 ± 38.84		HC	73.36 ± 7.78
Height (m)	mTBI	1.76 ± 0.11	Pulse rate (beats/min)	mTBI	70.81 ± 11.57
	HC	1.77 ± 0.09		HC	72.72 ± 14.23
BMI (m/kg^2^)	mTBI	28.32 ± 7.87	Respiration (breaths/s)	mTBI	14.17 ± 4.18
	HC	29.74 ± 11.49		HC	16.92 ± 11.45

Of the 36 mTBIs, 11 were associated with sports injuries, 9 with motor vehicle accidents, 8 with falls, 5 with other concussive events, 2 were not specified, and 1 with an improvised explosive device. Of these, 19 were associated with a loss of consciousness reported to last on average about 3 min, and 5 were associated with amnesia reported to last on average about 45 min. The average estimated age at the time of trauma was 23 years with a SD of 7 years, and the average estimated interval between trauma and testing was 3.3 years with a SD of 2.8 years (11).

### Instrumentation

#### Reduced Oxygen Breathing Device-2 with Integrated Pulse Oximeter

The NH stress was generated using the Reduced Oxygen Breathing Device (ROBD)-2 (Environics, Inc., Tollande, CT, USA), a commercial, off-the-shelf, portable, computerized, gas-blending instrument that mixes N_2_ with MSL air to produce breathable air with oxygen partial pressures comparable to oxygen partial pressures encountered at known altitudes (25–28). These air–nitrogen mixes are precise and repeatable and are used to induce NH safely, without risk of barotrauma or decompression sickness (Bends), and without using bagged mixed gases, which can be exhausted before all testing procedures have been completed. The ROBD instrumentation permits extensive laboratory testing without the complications associated with altitude chambers. In addition to MSL air, the ROBD generated three levels of NH stress. One was with a 15.5% O_2_ balanced with 84.5% N_2_. The second was a 14% O_2_ balanced with 86% N_2_. The third was a 13% O_2_ balanced with 87% N_2_. These approximated the percent O_2_ encountered at 8,000, 12,000 and 14,000 ft above MSL, respectively.

The ROBD includes a built-in conventional pulse oximeter to monitor SpO_2_ and pulse rate at either the subject’s finger or ear lobe. SpO_2_ and pulse rate were recorded once every minute of testing throughout the entire test sequence.

#### Functional Impairment Tester

The FIT (PMI, Inc., Rockville, MD, USA) is a commercial, off-the-shelf, screening device designed to assess oculometrics (i.e., eye movements and pupillometry) as a sign of neurological changes associated with drugs, alcohol, sleepiness, or other neurological deficits that express themselves in the reflexive oculomotor behavior of the eye (17, 18, 20, 29).

The FIT records the saccadic velocity of the eye as it shifts gaze between a pair of alternately flashing lights. The FIT also records pupil diameter in the dark, the latency of the pupil’s response to a flash of light, and the amplitude of that response, which is the difference between the pupil’s diameter in the dark and pupil’s peak constriction to the light flash. These four FIT measurements are made consecutively and require approximately a minute to complete. The FIT makes these measurements by analyzing infrared video images of the eye’s cornea, lens, and pupil.

The FIT was administered twice for each of the experimental conditions. The first FIT administration started 1 min into the experimental condition; the second FIT administration started after the psychometric evaluations had been completed for that experimental condition, which was on average about 13 min after the condition began. The results reported here are of the second FIT administration.

### Procedure

Individuals who responded to recruitment flyers were initially screened by phone. Potential study participants who met the inclusion criteria attended a single test session. During the test session, subjects first underwent the informed consent process. After consenting to participate in the study, subjects were medically screened by the study physician to verify self-reported health status. Female subjects provided a urine sample for pregnancy testing. The study physician ensured compliance with all inclusion/exclusion medical criteria.

After medical screening, the subject completed a set of practice FIT measures and a battery of psychometric evaluations before being connected to the ROBD. Five successful FIT practice trials were completed to ensure that the subject understood how the test worked and to make alignment adjustments to ensure that the subject was comfortable. The first three of these five FIT practice trials were completed without the subject being connected to the ROBD. For the last two FIT practice trials, the subject was fitted with the finger pulse oximeter sensor and the ROBD respirator (11). Once the correct sized respirator was securely attached to the face, the respirator was checked for leakage. If there was leakage or if the respirator did not fit or was uncomfortable, adjustments were made to fit the respirator properly. The fifth and last FIT warm-up trial was conducted with the ROBD turned on but not connected to the respirator; in this way, the subject would know what noises to expect during testing. Following these five FIT practice trials, formal testing began. Subjects were instructed to breathe normally. They were watched to ensure normal breathing and to guard against hypoventilation, hyperventilation, and hypocapnia. Every subject went through the same sequence of five conditions: 21% O_2_ Baseline, 15.5% O_2_, 14% O_2_, 13% O_2_, and 21% O_2_ Post Stress. The volunteer acclimated to each condition for 1 min before beginning the following sequence of testing: (1) FIT measures, (2) psychometric battery, and (3) a second set of FIT measures. The results of the psychometric battery were reported previously (10).

When the testing sequence was completed for each condition, the subject was returned to 21% O_2_ and allowed to take a break; if desired, the mask was removed for the volunteer to breathe normal room air. When the volunteer was ready to continue, ROBD airflow was resumed and the subject again donned the mask, which was checked to make sure the seal was tight. If the volunteer opted to continue testing without a break, the volunteer rested at 21% O_2_ for at least 1 min before exposure to the next condition. This procedure was repeated for each of the five breathing conditions. After the subject completed all five conditions, the subject was observed in the laboratory for at least 30 min to ensure that there were no signs of aftereffects of the hypoxic experience. These procedures took approximately 4 h to complete. All subjects were tested one at a time, and testing required two laboratory technicians. It may be noted that although the laboratory technicians knew whether the specific subject was an mTBI or HC volunteer, the technicians had no knowledge of the hypothesis being investigated.

For each subject, the duration of a condition depended on the length of time the subject needed to complete the scheduled measurements for the condition. Table 2 presents the average time (in minutes) needed to complete the measurements for each of the five conditions, the two groups of subjects, and for all subjects combined. An ANOVA showed that there were no statistically significant differences between subject groups or conditions.

**Table 2:** **Mean time (minutes) to complete measurements for each condition**.

Group	21% O_2_ baseline	15.5% O_2_	14% O_2_	13% O_2_	21% O_2_ post stress	Total^a^
mTBI	14.11	13.70	14.20	13.90	13.90	84.70
HC	13.86	14.50	14.20	13.40	13.60	83.10
Total mean	14.90	14.10	14.20	13.70	13.80	83.90

*^a^Note that the column named Total refers to the duration of the whole data collection session, which included rest breaks between tested conditions; consequently, the total durations are longer than the sum of the durations of the five conditions*.

### Statistical Analysis

#### Data Reduction

SpO_2_ and pulse rate were recorded by hand once a minute onto paper data collection sheets and subsequently entered into a computer for analyses. The FIT data were downloaded from the FIT software into a computer database and organized for analyses. All statistical analyses were performed with SPSS Statistics 20 (IBM Corp., Armonk, NY, USA). Alpha was set at the 0.05 level for all statistical evaluations.

#### Multiple Analyses of Variance

Standard multivariate analysis techniques were used to evaluate statistical significance of the results in order to control possible correlational dependencies among the numerous response parameters. Three Multiple Analyses of Variance (MANOVAs) were conducted. The first MANOVA (Table 3) simultaneously evaluated the influence of three independent variables on the dependent variables of SpO_2_ and pulse rate. The three independent variables were the between-subjects variable group (mTBI vs. HC), the within-subjects variable of the five percent O_2_ conditions, and the within-subjects variable time (the first and the last minute of the NH exposure). For the second MANOVA (Table 5), a linear regression (see next section) was first calculated as a function of the exposure time for each NH condition separately for the two subject groups. These regression calculations produced an intercept and slope for both subject groups for each of the five conditions. The MANOVA then evaluated whether these intercepts and slopes differed between groups (mTBI vs. HC) and the five percent O_2_ conditions. The third MANOVA (Table 7) simultaneously evaluated the influence of two independent variables, namely, group (mTBI vs. HC) and the five percent O_2_ conditions, on the four oculometric FIT response variables of Pupil Diameter, Pupil Constriction Amplitude, Pupil Constriction Latency, and Saccadic Velocity.

**Table 3:** **MANOVA: 2 (group) × 5 (percent O_2_) × 2 (time) for SpO_2_ and pulse rate**.

Effect	Test statistic	Degrees of freedom	*F*	*p*	η^2^
Group main effect	Wilks’ λ = 0.880	2, 69	14.85	<0.01	0.123
Percent O_2_ main effect	Wilks’ λ = 0.079	8, 63	91.71	<0.01	0.921
Time main effect	Wilks’ λ = 0.152	8, 69	192.96	<0.01	0.848
Group by percent O_2_ interaction effect	Wilks’ λ = 0.820	8, 63	1.73	0.11	0.180
Group by time interaction effect	Wilks’ λ = 0.859	8, 69	5.66	<0.01	0.141
Percent O_2_ by time interaction effect	Wilks’ λ = 0.104	8, 63	67.51	<0.01	0.896
Group by percent O_2_ by time interaction effect	Wilks’ λ = 0.667	8, 63	9.94	<0.01	0.333

As is standard practice with such multivariate procedures, Box’s *M* test was calculated to assess the equality of the covariance matrices associated with each of the MANOVAs. If Box’s *M* test was not significant, the covariance matrices were considered equivalent, and the significance of the differences in the MANOVA was evaluated with the Wilk’s λ statistic. If Box’s *M* was significant, the covariance matrices were deemed different, and the significance of the differences in the MANOVA was evaluated with Pillai’s trace.

For the MANOVAs reported in Tables 3, 5 and 7, univariate follow-up evaluations are reported in Tables 4, 6 and 8, respectively, along with the group by condition average and SEM. When univariate interactions between group and the other independent variables emerged significant, planned comparisons were performed to evaluate group differences for each of the three NH stress conditions while ensuring that group similarities existed at Baseline and Post Stress conditions. In the presence of such interactions, main effects were not further evaluated because between group differences were the focus of this study. Additionally, appropriate effect sizes are reported with each analysis, η^2^ for analyses of variance and Cohen’s *d* for *t*-tests.

**Table 4:** **Means and SEM by subject group for the first and last minute of each percent O_2_ condition**.

Time (exposure minute)	21% O_2_ baseline		15.5% O_2_		14% O_2_		13% O_2_		21% O_2_ post stress	
	First	Last	First	Last	First	Last	First	Last	First	Last
**SpO**_2_	Group *p* < 0.001, Percent O_2_ *p* < 0.001, Time *p* < 0.001, Group × Percent O_2_ *p* < 0.001, Group × Time *p* < 0.001, Percent O_2_ × Time *p* < 0.001, Group × Percent O_2_ × Time *p* < 0.001**									
mTBI	97.25 ± 0.08^†^	97.14 ± 0.10	96.08 ± 0.23	93.31 ± 0.20^†^^,^^‡^	95.08 ± 0.41	88.19 ± 0.37^†^^,^^‡^	94.39 ± 0.58	83.75 ± 0.91^†^^,^^‡^	96.14 ± 0.41	97.90 ± 0.03
HC	96.97 ± 0.08^†^	97.00 ± 0.10	96.39 ± 0.20	92.31 ± 0.27^†^^,^^‡^	94.83 ± 0.33	83.91 ± 0.74^†^^,^^‡^	93.83 ± 0.58	80.81 ± 0.91^†^^,^^‡^	95.81 ± 0.41	96.97 ± 0.12
**Pulse rate**	Group *p* = 0.317, Percent O_2_ *p* < 0.001, Time *p* < 0.001, Group × Percent O_2_ *p* = 0.50, Group × Time *p* = 0.06, Percent O_2_ × Time *p* < 0.001, Group × Percent O_2_ × Time *p* < 0.001**									
mTBI	72.19 ± 1.79	74.03 ± 1.58	74.61 ± 1.65	74.47 ± 1.89	72.83 ± 1.72	76.44 ± 1.76^†^^,^^‡^	70.81 ± 2.00	76.94 ± 1.92^†^	67.00 ± 1.85	70.44 ± 2.29
HC	75.94 ± 1.96	77.19 ± 1.95	75.53 ± 2.39	78.03 ± 2.16	73.47 ± 2.23	82.44 ± 2.13^†^^,^^‡^	72.33 ± 2.27	82.36 ± 2.30^†^	68.42 ± 2.14	71.56 ± 2.50

**Greenhouse–Geisser correction was used in the univariate ANOVA follow-ups to account for significant Mauchly’s test of sphericity with Epsilon values < 0.75. Overall, SpO_2_ results were stronger than pulse rate results. Main effects and two-way interactions were not considered important because mTBI and HC were matched pairs and the Baseline, first minutes of each hypoxic stress, and Post Stress conditions were all expected to be equivalent. Follow-up t-tests showed the groups only differed from each other during the last minute of each hypoxic stress for SpO_2_ but not for pulse rate.

*^†^Significantly different from the last minute of Baseline (*p* < 0.05)*.

*^‡^Significant group difference at the specified NH stress condition (*p* < 0.05)*.

#### Procedures Used to Determine the Linear Fit Analyses

**Figure 1:**
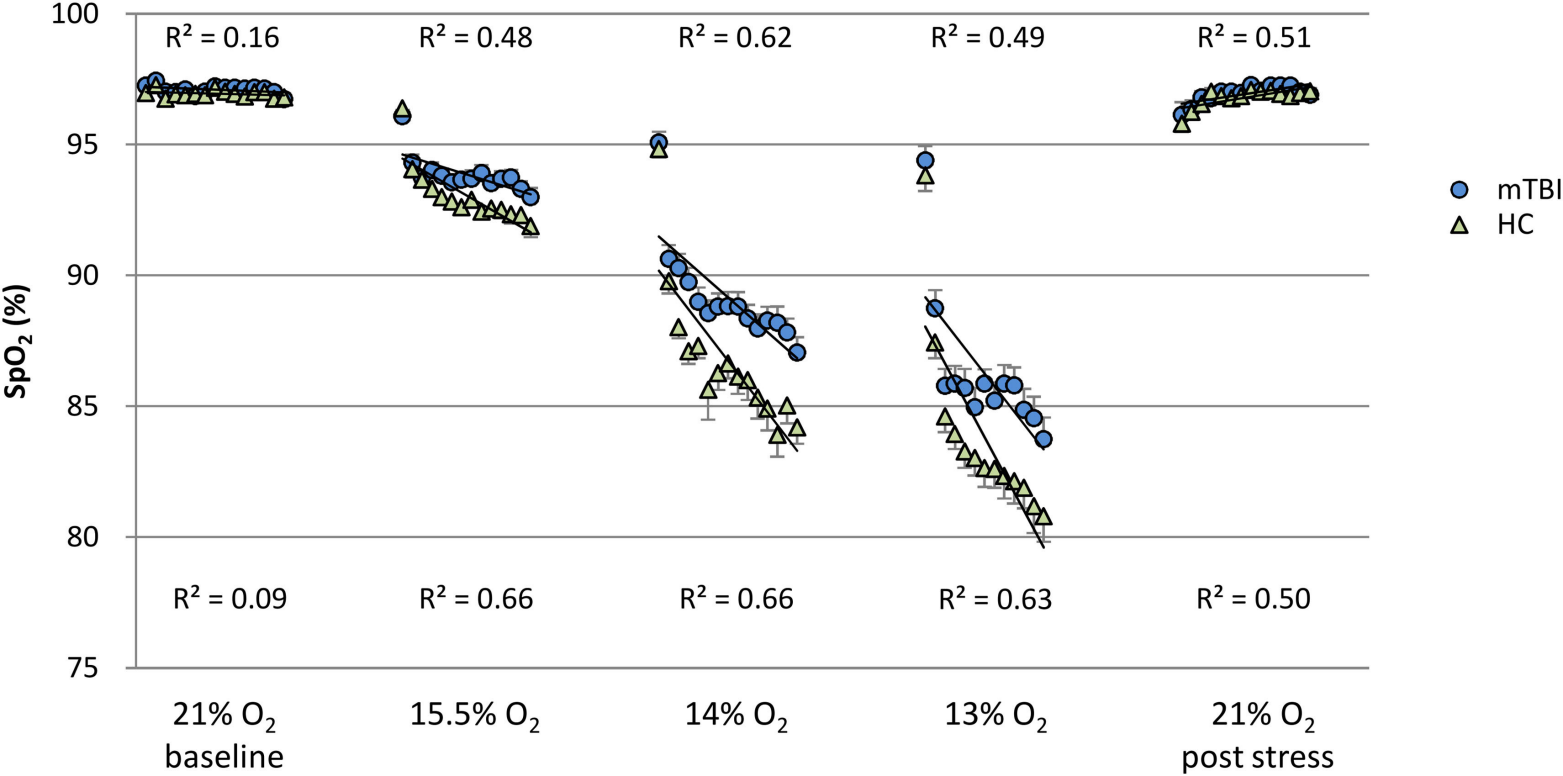
**SpO_2_: minute by minute group average and SEM for each percent O_2_ condition**. Note: R^2^ values are for the linear line of best fit for each group at each percent O_2_ condition.

**Figure 2:**
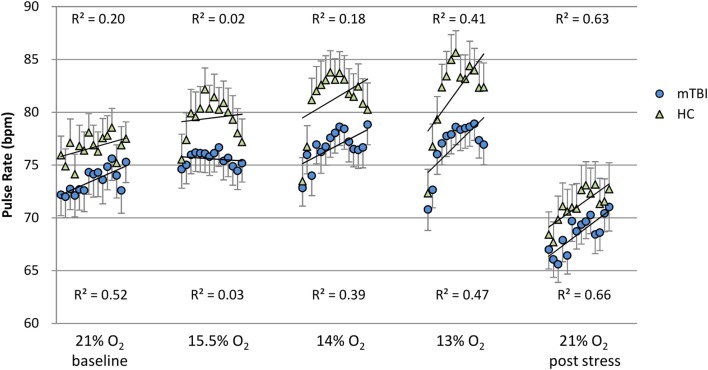
**Pulse rate: minute by minute group average and SEM for each percent O_2_ condition**. Note: R^2^ values are for the linear line of best fit for each group at each percent O_2_ condition.

To evaluate group differences over time for SpO_2_ and pulse rate, the linear line of best fit was determined for each subject in each percent O_2_ condition for the duration of that condition, but with the first minute excluded from the calculations. These individual slopes and intercepts were averaged over mTBI volunteers and over HC volunteers to generate a group average (±SEM) slope and a group average (±SEM) intercept for SpO_2_ and pulse rate. These average regression slopes and intercepts are presented in Table 6. Figures 1 and 2 depict the average regression lines and the data, including first and last minute of each condition by group. These values were used in the 2 (group) × 5 (percent O_2_) MANOVA (Table 5) to evaluate the slope and intercept differences between groups.

**Table 5:** **MANOVA: 2 (group) × 5 (percent O_2_) for slope and intercept of SpO_2_ and pulse rate**.

Effect	Test statistic	Degrees of freedom	*F*	*p*	η^2^
Group main effect	Pillai’s trace = 0.178	4, 67	3.64	<0.01	0.178
Percent O_2_ main effect	Pillai’s trace = 0.955	16, 55	72.53	<0.01	0.955
Group by percent O_2_ interaction effect	Pillai’s trace = 0.261	16, 55	1.22	0.29	0.001

#### FIT Correlation with SpO_2_ and Pulse Rate

Simple bivariate correlations among the four FIT measures and the two pulse oximeter measures, SpO_2_ and pulse rate, were determined by group (Table 9).

## Results

Figures 1 and 2 show the group average response to each percent O_2_ condition for each minute of exposure. These figures illustrate the average group response to reduced O_2_ over the period of exposure. Average SpO_2_ (Figure 1) for the two groups was the same at the Baseline and the Post Stress conditions. At the beginning of each NH stress condition, SpO_2_ for the mTBI and HC groups was indistinguishable; but with continued hypoxic exposure, SpO_2_ decreased for both groups, decreasing more for the HC group than the mTBI group. The pulse rate response (Figure 2) tended to increase for both groups from the Baseline condition through the 13% O_2_ condition, but a marked drop in pulse rate is evident at the conclusion of the hypoxic exposures, as reflected in the pulse rate recorded at Post Stress.

### MANOVA of SpO_2_ and Pulse Rate

The 2 (group) × 5 (percent O_2_) × 2 (time) MANOVA (Table 3) was performed to test differences between the mTBI and HC groups at the first and last minute of each condition for the dependent measures, SpO_2_ and pulse rate. Box’s *M* test of equality of covariance matrices was not significant [*M* = 328.15, *F*(210, 14,975) = 1.08, *p* = 0.194]. Table 3 shows that six of the seven effects were significant.

Results of follow up univariate tests are provided in Table 4. To account for significant Mauchly’s test of sphericity, Greenhouse–Geisser corrected univariate tests (e.g., χ^2^ = 142.94, df = 9, *p* < 0.001), showed the three-way interaction for group by percent O_2_ by time was significant for both SpO_2_ [*F*(2.17,151.8) = 5.29, *p* < 0.001, η^2^ = 0.852] and pulse rate [*F*(3.21,224.8) = 3.05, *p* < 0.001, η^2^ = 0.733]. Follow-up planned comparisons showed mTBI and HC were not statistically different at the first and last minute of each 21% O_2_ condition and the first minute of the three NH stress conditions for both SpO_2_ [*F*(2.93,204.92) = 0.399, *p* = 0.749, η^2^ = 0.006] and pulse rate [*F*(4.83,338.25) = 0.598, *p* = 0.696, η^2^ = 0.008]. These non-significant results indicate that both groups were indistinguishable as they began each NH stress condition, and that NH did not have differential lingering aftereffects on the two subject groups.

Further follow-up compared Baseline to the last minute of the three hypoxic stress conditions using *t*-tests to identify group differences within conditions (Table 4). SpO_2_ was significantly different from Baseline during the last minute of all three reduced percent O_2_ stress conditions [*F*(1.84,128.68) = 6.92, *p* = 0.002, η^2^ = 0.090], with mTBI evidencing significantly higher SpO_2_ than the controls during the last minute of each condition, specifically 15.5% O_2_ [*t*(70) = 2.55, *p* = 0.013, *d* = 0.481], 14% O_2_ [*t*(70) = 4.09, *p* < 0.001, *d* = 0.834], and 13% O_2_ [*t*(70) = 2.29, *p* = 0.025, *d* = 0.458]. For pulse rate, differences between baseline and the last minute of each stress approached significance [*F*(2.66,186.29) = 2.74, *p* = 0.052, η^2^ = 0.038], and group pulse rate differences only emerged during the last minute of 14% O_2_ and approached significances at 13% O_2_; specifically 15.5% O_2_ [*t*(70) = 1.24, *p* = 0.218, *d* = 0.253], 14% O_2_ [*t*(70) = 2.17, *p* = 0.034, *d* = 0.511], and 13% O_2_ [*t*(70) = 1.81, *p* = 0.075, *d* = 0.362].

### MANOVA of SpO_2_ and Pulse Rate Slope and Intercept

A 2 (group) × 5 (percent O_2_) MANOVA (Table 5) evaluated the slope and intercept of the SpO_2_ and pulse rate responses throughout each condition. Box’s *M* test of equality of covariance matrices was significant [*M* = 365.36, *F*(210, 14,975) = 1.21, *p* = 0.022], which indicated that the assumption of equivalent covariance across groups was not met; consequently, Pillai’s trace was used to control for Type-I error. Table 5 shows that the main effect of group and of percent O_2_ condition was significant, but there was no interaction between these variables. The main effects emerged significant but the group by percent O_2_ interaction was not.

Follow-up test results are provided in Table 6. Follow-up planned comparisons showed the groups were not statistically different at the two 21% O_2_ conditions for SpO_2_ slope [*F*(1,70) = 0.091, *p* = 0.764, η^2^ = 0.001], SpO_2_ intercept [*F*(1,70) = 0.057, *p* = 0.812, η^2^ = 0.001], pulse rate slope [*F*(1,70) = 0.003, *p* = 0.956, η^2^ = 0.001], and pulse rate intercept [*F*(1,70) = 0.079, *p* = 0.780, η^2^ = 0.001]. These non-significant results indicated that both groups were initially indistinguishable and did not differentially experience lingering effects of NH. These results also show that the main effects for percent O_2_ reported in Table 6 are not attributable to differences at the two equivalent conditions of Baseline and Post Stress.

**Table 6:** **Means and SEM by subject group for SpO_2_ and pulse rate regression slopes and intercepts**.

	21% O_2_ baseline	15.5% O_2_	14% O_2_	13% O_2_	21% O_2_ post stress
**SpO_2_ slope**	Group *p* < 0.001, Percent O_2_ *p* < 0.001, Group × Percent O_2_ *p* < 0.001**				
mTBI	−0.01 ± 0.01	−0.06 ± 0.02^†^^,^^‡^	−0.21 ± 0.03^†^^,^^‡^	−0.25 ± 0.05^†^^,^^‡^	+0.04 ± 0.02
HC	−0.01 ± 0.01	−0.14 ± 0.02^†^^,^^‡^	−0.34 ± 0.07^†^^,^^‡^	−0.44 ± 0.07^†^^,^^‡^	+0.04 ± 0.02
**SpO_2_ intercept**	Group *p* = 0.097, Percent O_2_ *p* < 0.001, Group × Percent O_2_ *p* = 0.12**				
mTBI	97.2 ± 0.09	94.1 ± 0.28	94.1 ± 0.28^†^	87.4 ± 0.57	96.6 ± 0.21
HC	97.0 ± 0.10	93.9 ± 0.24	93.9 ± 0.24^†^	86.3 ± 0.46	96.5 ± 0.24
**Pulse rate slope**	Group *p* = 0.709, Percent O_2_ *p* < 0.001, Group × Percent O_2_ *p* = 0.82**				
mTBI	0.19 ± 0.07	−0.07 ± 0.09	0.15 ± 0.08	0.27 ± 0.09^†^	0.36 ± 0.07
HC	0.13 ± 0.08	−0.08 ± 0.10	0.07 ± 0.10	0.34 ± 0.10^†^	0.29 ± 0.09
**Pulse rate intercept**	Group *p* = 0.131, Percent O_2_ *p* < 0.001, Group × Percent O_2_ *p* = 0.73**				
mTBI	71.9 ± 1.80	76.2 ± 1.90	75.7 ± 1.80^†^	75.3 ± 1.90^†^	65.7 ± 1.80
HC	75.6 ± 1.90	80.4 ± 2.20	81.3 ± 2.00^†^	80.1 ± 2.10^†^	69.0 ± 2.00

**Greenhouse–Geisser correction was used in the univariate ANOVA follow-ups to account for significant Mauchly’s test of sphericity with Epsilon values < 0.75. Overall, SpO_2_ results were stronger than the pulse rate results and only relevant interactions effects are identified. Main effects were not considered important because mTBI and HC were matched pairs and the Baseline and Post stress were expected to be equivalent. Follow-up t-tests for the significant SpO_2_ interaction show the mTBI group had shallower slopes than the HCs and both groups had steeper slopes than the 21% O_2_ conditions.

*^†^Significantly different from Baseline (*p* < 0.05)*.

*^‡^Significant group difference at the specified percent O_2_ condition (*p* < 0.05)*.

Although the omnibus group by percent O_2_ interaction failed to reach significance, univariate follow-up tests, using Greenhouse–Geisser correction to account for a significant Mauchly’s test of sphericity, showed that the non-significant MANOVA masked a single significant interaction for SpO_2_ slope [*F*(2.3,161.3) = 2.863, *p* < 0.001, η^2^ = 0.569] (see Table 6). Follow-up *t*-tests for SpO_2_ slope revealed the mTBI group had shallower slopes than the HC group for each NH stress condition, specifically 15.5% O_2_ [*t*(70) = 3.09, *p* = 0.003, *d* = 0.564], 14% O_2_ [*t*(70) = 2.23, *p* = 0.029, *d* = 0.482], and 13% O_2_ [*t*(70) = 2.27, *p* = 0.026, *d* = 0.456].

### Oculometrics

#### MANOVA FIT Outcome Measures

A 2 (group) × 5 (percent O_2_) MANOVA evaluated the four FIT outcome measures (pupil diameter, pupil constriction amplitude, pupil latency, and saccadic velocity). Box’s *M* test of equality of covariance matrices was not significant [*M* = 361.95, *F*(210, 11,650) = 1.123, *p* = 0.109], indicating the assumption of equivalent covariance across groups was not violated. The MANOVA for FIT outcomes emerged with a significant percent O_2_ effect (Table 7), showing an effect of NH on the FIT measures, an effect that was not different between subject groups and thus has little bearing on the purposes of the present experiment. Confirming the non-significant omnibus interaction, none of the univariate interaction effects emerged significant (see Table 8).

**Table 7:** **MANOVA: 2 (group) × 5 (percent O_2_) for FIT outcome measures**.

Effect	Test statistic	Degrees of freedom	*F*	*p*	η^2^
Group main effect	Wilks’ λ = 0.893	4, 59	1.76	0.19	0.107
Percent O_2_ main effect	Wilks’ λ = 0.365	16, 47	5.11	<0.01	0.635
Group by percent O_2_ interaction effect	Wilks’ λ = 0.730	16, 47	1.09	0.39	0.270

**Table 8:** **Group by condition means and SEM for each of the four FIT-dependent measures**.

	21% O_2_ baseline	15.5% O_2_	14% O_2_	13% O_2_	21% O_2_ post stress
**Pupil diameter**	Group *p* = 0.066, Percent O_2_ *p* < 0.001, Group × Percent O_2_ *p* = 0.617**				
mTBI	5.42 ± 0.46	5.29 ± 0.16	5.17 ± 0.16	5.08 ± 0.17	5.19 ± 0.15
HC	5.87 ± 0.16	5.68 ± 0.16	5.59 ± 0.16	5.43 ± 0.17	5.66 ± 0.16
**Pupil constriction amplitude**	Group *p* = 0.618, Percent O_2_ *p* < 0.001, Group × Percent O_2_ *p* = 0.391**				
mTBI	1.13 ± 0.04	1.12 ± 0.04	1.10 ± 0.04	1.06 ± 0.04	1.11 ± 0.04
HC	1.19 ± 0.04	1.14 ± 0.04	1.10 ± 0.04	1.06 ± 0.04	1.16 ± 0.04
**Pupil constriction latency**	Group *p* = 0.252, Percent O_2_ *p* = 0.276, Group × Percent O_2_ *p* = 0.680**				
mTBI (ms)	299.97 ± 4.04	298.28 ± 3.82	301.27 ± 3.87	299.85 ± 3.91	303.02 ± 4.10
HC (ms)	294.22 ± 4.17	293.71 ± 3.94	295.24 ± 4.00	293.57 ± 4.04	294.44 ± 4.24
**Saccadic velocity**	Group *p* = 0.685, Percent O_2_ *p* < 0.001, Group × Percent O_2_ *p* = 0.091**				
mTBI (ms)	74.25 ± 1.21	72.47 ± 1.29	72.96 ± 1.18	72.06 ± 1.33	71.67 ± 1.34
HC (ms)	72.32 ± 1.25	73.64 ± 1.33	71.99 ± 1.22	71.01 ± 1.37	71.02 ± 1.38

**Greenhouse–Geisser correction was used in the univariate ANOVA follow-ups to account for significant Mauchly’s test of sphericity with Epsilon values < 0.75. Main effects were not considered important because mTBI and HC were matched pairs and the Baseline and Post Stress conditions were expected to be equivalent. None of the interactions emerged significant.

#### Correlation of FIT Outcomes with SpO_2_ and Pulse Rate

Simple bivariate correlations among the FIT outcome measures and SpO_2_ and pulse rate were determined for both groups (Table 9). Although four relationships emerged significant, Pearson’s *r* indicated weak relationships.

**Table 9:** **Correlation among FIT outcome measures and SpO_2_ and pulse rate**.

Group	Pupil diameter		Pupil constriction amplitude		Pupil constriction latency		Saccadic velocity	
	*r*	*p*	*r*	*p*	*r*	*p*	*r*	*p*
**SpO_2_**								
mTBI	0.108	0.15	0.105	0.17	−0.003	0.97	0.118	0.11
HC	0.169*	0.02	0.250*	0.01	−0.076	0.32	0.076	0.31
**Pulse rate**								
mTBI	−0.071	0.35	−0.016	0.833	0.036	0.637	−0.123	0.10
HC	0.288*	0.01	−0.027	0.72	0.087	0.25	0.170*	0.02

*Significant p < 0.05.

## Discussion

The three levels of NH stress used in this study produced SpO_2_ and pulse rate responses that differed between the mTBI and matched HC groups. However, SpO_2_ and pulse rate were indistinguishable between mTBI and HC groups during Baseline, at the initiation of each NH stress condition, and during the Post NH Stress condition. Thus, consistent with previous stress and mTBI research involving physiological metrics, the mTBI group was not different from the matched HC group when not stressed.

In mammals, SpO_2_ normally decreases with reduced inspired O_2_ concentrations. The findings reported here showed the mTBI group had SpO_2_ values that were consistently higher, i.e., less reduced, than the HCs throughout each NH stress condition. Furthermore, at a constant percent O_2_, the falloff in SpO_2_ over time was less for the mTBI than for the HC group (Figure 1). These findings, the first to compare SpO_2_ between mTBI and HC groups under NH in humans, may seem counterintuitive. However, it may be noted that a normal response to hypoxia is an increase in ventilation, the hypoxic hyperventilatory response (HVR). An increased HVR in the mTBI group could account for the increase in SpO_2_ relative to the HC group and such a differential effect on the HVR may reflect changes associated with mTBI in the autonomic and cardiovascular systems (30). Furthermore, the observation that pulse rate trended lower in the hypoxic mTBI subjects than in hypoxic HC would also be consistent with an increased HVR in the mTBI group. The report by Len et al. (5) that PETCO_2_ is lower in the mTBI group than HC group when breath holding and when hyperventilating seems consistent with this idea. Unfortunately, a broader set of respiration measures (e.g., ETO_2_, respiration rate, minute ventilation, and total volume of gas expired per condition) was not collected in either the present study nor reported by Len et al. Exploring the relationships among blood oxygenation and respiration in those with a history of mTBI who are currently asymptomatic and appear to have fully recovered from injury is necessary to further explore the latent impact of brain injury.

On the other hand, the SpO_2_ differences reported here could possibly occur because of differences in physical fitness between the two subject groups, with the mTBI group being more physically fit than the HC group. However, this seems unlikely since the HCs were selected to ensure the groups did not differ statistically along the dimensions of age, height, BMI, systolic and diastolic blood pressure, pulse rate, or respiration rate; moreover, the pulse rate response was not significantly different between groups.

It may be noted that the pulse rate recorded during Post Stress (Figure 2), while not statistically different between the mTBI and HC groups, was statistically significantly less than the pulse rate recorded during the Baseline, despite the fact that neither condition posed any hypoxic stress. This significant decrease in pulse rate immediately following the conclusion of the 13% O_2_ is consistent with previous findings reported in the literature. Roche et al. (31) measured cardiovascular response in a group of healthy volunteers before, during, and after a 15-min hypoxic exposure with 11% O_2_ stress and reported that heart rate increased 19% over the initial baseline during hypoxia; but more interesting, during the 15-min recovery period heart rate decreased 10% below baseline. The methods used by Roche et al., which included measures of HRV, assessed the autonomic nervous system and pointed to an exaggerated sympathetic response to NH followed by an enhanced parasympathetic response during the recovery period, which is completely consistent with the findings reported here.

The FIT showed sensitivity to hypoxia as noted in Table 8; the main effects for NH stress were significant for diameter and response amplitude of the pupil and saccadic velocity. The main effect for NH stress was consistent with the study by Cymerman et al. (17), which showed changes in pupil diameter, response amplitude, and latency but without changes in saccadic velocity following 3 h of continuous exposure to an altitude of 4,300 m (14,107 ft). However, since the FIT failed to show differences between the mTBI and HC groups it is possible that there are, in fact, no differences in the ocular motor behavior between the two subject groups; but based on the emerging scientific literature describing oculometrics in mTBI (12, 22, 32–34), it seems more likely that the FIT was merely insufficiently sensitive to differences that do exist. It should be noted that the FIT was designed as a non-invasive, oculomotor, performance-based fitness for duty impairment screener, and as such, the FIT may simply have been inadequate for the purposes of this research.

Normobaric hypoxia did not produce undesirable physiological outcomes in the mTBI subjects in this study. SpO_2_ and pulse rate values did change with increased NH; however, values did not approach limits considered to be dangerous to health. Also, no trial had to be stopped due to health concerns or extreme discomfort experienced by the subjects. Unlike the breathing behavior alterations used by Len et al. (5, 6), the ROBD produces a sustained physiological stress and affords the opportunity to administer additional tests under a range of constant NH stress levels. These effects were reversed and disappeared when the subjects were returned to sea level and room air. Our results are consistent with the findings reported by Ewing et al.; individuals with a history of mTBI may be particularly susceptible to the effects of mild or moderate hypoxia. The hypobaric hypoxic research paradigm that Ewing et al. (9) used requires a significant infrastructure to support. The NH method used here is portable, and by comparison with the hypobaric paradigm, is safe, inexpensive, and easy to create in most laboratory or clinical settings.

It is important to acknowledge limitations of the present study. One important limitation was the wide range of the time between a volunteer’s trauma and test participation, a range from 1.7 to 119.7 months. The elapsed time since injury was not controlled. Second, physical activity level (e.g., hours per week) or fitness level (VO_2_ max) were not used in the matching criteria between subjects. Third, the pulse oximeter data were very limited, recorded once a minute by hand. Finally, the history of brain injury was determined by self-report. It is possible that individual subjects were not accurate about their brain injury history, a limitation that could be present in both groups.

## Conclusion

When exposed to mild or moderate normobaric hypoxic stress conditions simulating altitudes up to 14,000 ft above MSL: (1) peripheral oxygen saturation (SpO_2_) differences emerged between the mTBI and matched HCs, (2) pulse rate trended lower in the mTBI group, and (3) FIT oculometrics were not sensitive to group differences. These findings demonstrate that a relatively minor hypoxic challenge can reveal measurable differences in SpO_2_ and pulse rate but not FIT measures, in otherwise asymptomatic individuals with a history of mTBI.

## Author Contributions

LT had overall responsibility of the project. PS was primary lead on data analysis and an equal contributor to manuscript writing. JB was an equal contributor on data analysis, interpretation, and manuscript writing.

## Disclaimer

The opinions, interpretations, conclusions, and recommendations are those of the authors and are not necessarily endorsed by the U.S. Army and/or the Department of Defense.

## Conflict of Interest Statement

The authors declare that the research was conducted in the absence of any commercial or financial relationships that could be construed as a potential conflict of interest.
